# Comparisons of retinal vessel diameter and glaucomatous parameters between both eyes of subjects with clinically unilateral pseudoexfoliation syndrome

**DOI:** 10.1371/journal.pone.0179663

**Published:** 2017-06-23

**Authors:** Yasuyuki Takai, Masaki Tanito, Tetsuro Omura, Ryo Kawasaki, Yumiko Kawasaki, Akihiro Ohira

**Affiliations:** 1 Department of Ophthalmology, Shimane University Faculty of Medicine, Izumo, Japan; 2 Division of Ophthalmology, Matsue Red Cross Hospital, Matsue, Japan; 3 Department of Public Health, Yamagata University Graduate School of Medical Science, Yamagata, Japan; Bascom Palmer Eye Institute, UNITED STATES

## Abstract

**Purpose:**

Pseudoexfoliation syndrome (PEX), the most common identifiable cause of open-angle glaucoma, might affect the retinal hemodynamics. To test this, we compared retinal vessel diameter and glaucoma-related parameters between eyes with pseudoexfoliation material (PE+) and fellow unaffected (PE-) eyes of patients with clinically unilateral PEX.

**Methods:**

The medical records of 30 consecutive Japanese subjects were reviewed retrospectively. The retinal vessel diameters were measured and expressed as the central retinal arteriolar equivalent (CRAE) and central retinal venular equivalent (CRVE) using standardized software. During the chart review, we recorded glaucoma-related parameters including intraocular pressure (IOP), visual field mean deviation (MD) value, planimetrically measured vertical cup-to-disc (C/D) ratio, circumpapillary retinal nerve fiber layer thickness (cpRNFLT) and macular inner retinal thickness (mIRT) measured by spectral-domain optical coherence tomography, anterior chamber flare (ACF), corneal endothelial cell density (CECD), and number of antiglaucoma medications.

**Results:**

Compared with PE- eyes, the CRAE, CRVE, MD, cpRNFLT and mIRT, and CECD were significantly lower in PE+ eyes; the IOP, vertical C/D ratio, number of antiglaucoma medications, and ACF were significantly higher in PE+ eyes (*P*<0.0001 for all comparisons). The CRAE, cpRNFLT, and MD were correlated positively with each other (ρ = 0.456–0.499, *P*<0.0001–0.0002) and negatively with the IOP (ρ = -0.562- —0.432, *P*<0.0001–0.0006). The vertical C/D ratio was correlated positively with the IOP (ρ = 0.483, *P*<0.0001) and negatively with the CRAE, cpRNFLT, and MD (ρ = -0.745–-0.479, *P*<0.0001–0.0001).

**Conclusions:**

Deposition of PE can cause retinal vessel narrowing in arterioles and venules. The roles and mechanisms of retinal vessel narrowing in glaucoma pathogenesis need clarification.

## Introduction

Pseudoexfoliation syndrome (PEX) is an age-related, generalized disorder of the extracellular matrix, the most common characteristics of which are production and progressive accumulation of fibrillar extracellular material in many ocular tissues[1]. The abnormal deposition of pseudoexfoliation material (PE) in the anterior segment causes a broad spectrum of ocular manifestations including intraocular pressure (IOP) elevation, cataract progression, phacodonesis, keratopathy, and blood-aqueous barrier dysfunction[2–4]. PEX is a major risk factor for open-angle glaucoma (OAG) worldwide, and approximately half of the cases present unilaterally[5]. In a study of patients with unilateral PEX, 32% progressed to glaucoma, with a mean conversion time of 2.8 years[6]. A clinical observational study of consecutive central retinal vein occlusion cases reported a high prevalence rate of PEX (22% in 15 eyes)[7]. The presence of PE was reported to be a likely risk factor for retinal vascular occlusive disorders[7–9], decreased ocular arterial blood flow velocities, and increased vascular resistance[10]. PE has been found in various ocular blood vessels, e.g., the walls of iris vessels, posterior ciliary arteries, vortex veins, and central retinal vessels[11, 12]. Thus, blood flow might be altered by PE because of increased permeability, narrowing, and ultimately obstruction of the retinal vessels[13, 14]. Previously, Mitchell et al. reported that eyes with OAG had significantly smaller retinal arteriolar diameters than normal eyes and eyes with ocular hypertension[15]. To clarify the association between PE deposition and retinal vessel narrowing, we compared the retinal vessel diameter and various glaucoma-related parameters in affected (PE+) and unaffected fellow (PE-) eyes of patients with clinically unilateral PEX.

## Materials and methods

The current study was part of the study protocol “Epidemiologic Study of Ocular Morphology and Function,” that the Ethics Committee of Shimane University Hospital approved and adhered with the tenets of the Declaration of Helsinki. The ethics committee waived the requirement for the patient’s informed consent regarding the use of their medical record data in accordance with the regulations of Japanese Guidelines for Epidemiologic Study issued by the Japanese Government, and instead, the protocol was posted at the outpatient clinic to notify the study to the participants. We reviewed retrospectively the medical records of 30 Japanese patients diagnosed with clinically unilateral PEX and recorded a full set of the measurement parameters recorded at Shimane University Hospital from May 2008 to August 2012. Regardless of having glaucoma or not, unilateral affected (PE+) eye was defined as unilateral presence of detectable PE at the pupillary border or on the anterior lens capsule, and unaffected fellow (PE-) eye was defined as no presence of detectable PE by slit-lamp observation under mydriasis. We excluded patients with ocular diseases except for glaucoma and cataract and those who had undergone previous ocular surgeries and laser treatment. We carefully excluded the uveitic eyes by reviewing the medical chart and recorded slit lamp and gonioscopic photographs. Color fundus photographs were obtained from all patients with excellent visibility of the optic disc margins and vessel borders using the Nonmyd WX fundus camera (Kowa, Nagoya, Japan). The best-corrected logarithm of the mimimum angle of resolution visual acuity (logMAR VA) was measured. The ocular axial lengths were measured by AL-3000 ultrasound biometry (Tomey, Nagoya, Japan). The IOP was measured by Goldmann applanation tonometry, and the visual field mean deviation (MD) values of the Swedish Interactive Threshold Algorithm-standard central 30–2 program were measured using the Humphrey Visual Field Analyzer (Carl Zeiss Humphrey, San Leandro, CA). The circumpapillary retinal nerve fiber layer thickness (cpRNFLT) and macular inner retinal thickness (mIRT) were measured using the RS-3000 Advance spectral-domain optical coherence tomography (Nidek, Gamagori, Japan) machine. To image the cpRNFLT, raster scanning over a 6 × 6-mm^2^ area centered on the optic disc center was performed at a scan density of 512 A-scans (horizontal) × 128 B-scans (vertical) using a 3.45-mm-diameter circle positioned automatically around the optic disc in each three-dimensional data set. The mean thickness on the 3.45-mm-diameter circle was recorded as the cpRNFLT in this study. For mIRT imaging, raster scanning over a 9 × 9-mm^2^ area centered on the foveal center was conducted at a scan density of 512 A-scans (horizontal) × 128 B-scans (vertical). The mIRT was measured between the internal limiting membrane and the outer boundary of the inner plexiform layer. The mean thickness within the 9-mm circle centered on the foveal center was recorded as the mIRT in this study[16]. The anterior chamber flare (ACF) was measured using the FM-600 laser flare meter (Kowa). The corneal endothelial cell density (CECD) was measured using the EM-3000 specular microscope (Tomey). The number of antiglaucoma medications used were recorded. All measurements were required to be recorded within 1-week period and the number of antiglaucoma medications used in this period was adopted. These parameters of both eyes were collected by chart review.

### Retinal vessel diameter and vertical cup-to-disc (C/D) ratio measurement on retinal photographs

**Fig 1:**
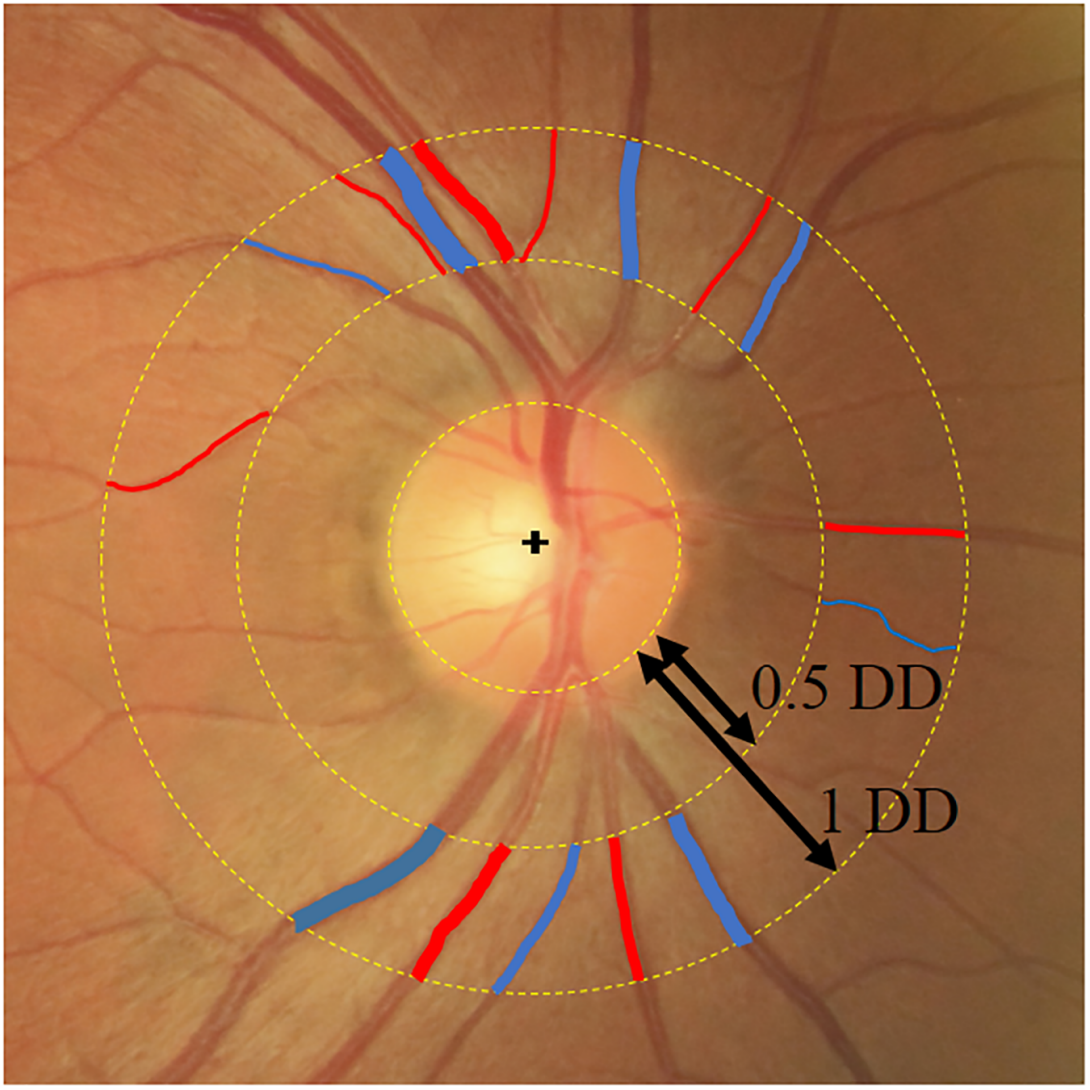
Measurement of the CRAE and CRVE using the IVAN software. All retinal arteries (red) and veins (blue) that pass completely through the circumferential zone 0.5 to 1 disc diameter from the optic disc margin are evaluated. The CRAE and CRVE are calculated using the revised Parr-Hubbard formulas. DD, disc diameter.

The retinal vessel diameters were measured on digitized 40-degree fundus photographs centered on the optic discs using the IVAN software developed by the Department of Ophthalmology and Visual Science, University of Wisconsin, Madison, the method for which has been described previously[17]. Briefly, all vessels were measured that were 25 microns or larger and passed completely through a circumferential zone 0.5 to 1 disc diameter from the optic disc margin[15]. A trained grader (YT) identified each vessel (Fig 1) as an arteriole or a venule. The six widest arteriolar and venular diameters then were defined as the central retinal arteriolar equivalent (CRAE) or the central retinal venular equivalent (CRVE) using the revised Parr-Hubbard formulas of Knudtson et al[18, 19]. The magnification of the optic media in an eye was corrected according to the method of Littmann, with consideration of the refractive error[20].

The detailed method for measuring the vertical C/D ratio using the CDSketch Software (Kowa, Tokyo, Japan) (downloadable from http://www.kowa.co.jp/e/life/app_download/) was described previously[21]. The software calculates automatically the parameters based on the contour lines of the optic disc margin and cup determined by the trained grader (YT).

Statistical analyses were performed using JMP version 11 (JMP Statistical Discovery, Cary, NC). The retinal vessel and glaucoma-related parameters were compared between the PE+ and PE- eyes using the Wilcoxon signed-rank test to test the null hypothesis that deposition of PE affects neither retinal vessel calibers nor any of glaucoma-related parameters. Correlations among the parameters were assessed using the Spearman’s rank correlation test to test the null hypothesis that retinal vessel calibers didn’t associate with any of glaucoma-related parameters. *P* < 0.01 was considered statistically significant.

## Results

Thirty adult subjects (18 females, mean age 73.1 ± 9.1 years) were recruited. Table 1 shows the comparisons of parameters between the PE+ and PE- eyes.

**Table 1:** Comparison of parameters between PE+ and PE- eyes.

	PE (+) (n = 30)	PE (-) (n = 30)	*P* value*
logMAR VA	0.30 ± 0.59	0.07 ± 0.18	< .0001
Axial length (mm)	23.88 ± 1.69	23.72 ± 1.66	0.0009
IOP (mmHg)	25.5 ± 8.28	16.6 ± 3.60	< .0001
MD (dB)	-15.34 ± 9.06	-4.01 ± 5.33	< .0001
vertical C/D ratio	0.76 ± 0.09	0.60 ± 0.08	< .0001
cpRNFLT (μm)	62.51 ± 17.7	89.55 ± 13.0	< .0001
mIRT (μm)	63.92 ± 17.2	84.85 ± 13.9	< .0001
CRAE (μm)	115.9 ± 10.8	125.4 ± 10.5	< .0001
CRVE (μm)	171.9 ± 14.5	179.5 ± 16.2	< .0001
ACF (photon counts/msec)	21.5 ± 29.0	9.5 ± 6.07	< .0001
CECD (cells/mm^2^)	2399 ± 205.0	2566 ± 246.7	< .0001
Antiglaucoma medications	2.32 ± 1.43	1.10 ± 1.35	< .0001

PE, pseudoexfoliation material; log MAR VA, logarithm of the minimum angle of resolution visual acuity; IOP, intraocular pressure; MD, visual field mean deviation values; vC/D ratio, vertical cup-to-disc ratio; cpRNFL, circumpapillary retinal nerve fiber layer; mIR, macular inner retinal; CRAE, central retinal arteriolar equivalent; CRVE, central retinal venular equivalent; ACF, anterior chamber flare; CECD, corneal endothelial cell density; Medications, number of antiglaucoma medications use.

*By the Wilcoxon signed-rank test.

Data are expressed as mean±standard deviation.

**Fig 2:**
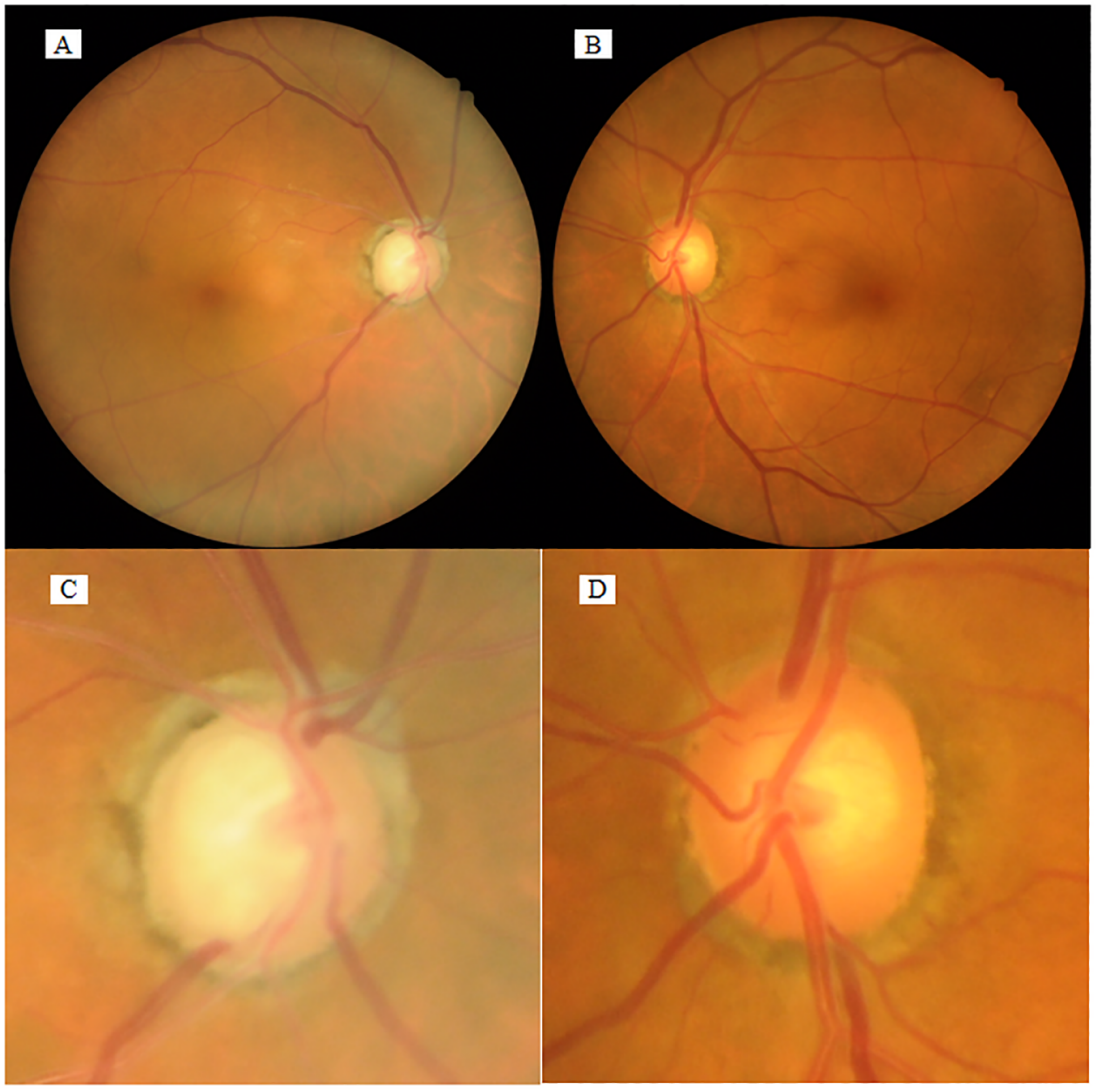
Fundus photographs of a unilateral PEX case. In this representative case of unilateral PEX, the fundus photographs show that the retinal artery diameter is narrower in the PE+ right eye (**A, C**) than in the PE- left eye (**B, D**). In this case, CRAE and CRVE are 121.3 and 165.5 microns, respectively, in the PE+ eye, and 127.9 and 181.4 microns, respectively, in the PE- eye. (**C, D**) Higher magnification of the optic disc in **A** and **B**, respectively. Magnifications, in **A** and **B** ×1; in **C** and **D** ×2.5.

Compared with the PE- eyes, the CRAE, CRVE, MD, cpRNFLT, mIRT, and CECD were significantly lower and the logMAR VA, IOP, vertical C/D ratio, number of medications, and ACF were significantly higher in PE+ eyes (*P*<0.0001 for all comparisons). In a representative case of unilateral PEX, retinal vessel narrowing was seen in the PE+ eye compared with the PE- eye (Fig 2).

Table 2 shows the correlations among the various parameters. The CRAE, cpRNFLT, and MD were correlated positively with each other (ρ = 0.456–0.499, *P*<0.0001–0.0002) and negatively with the IOP (ρ = -0.562–-0.431, *P*<0.0001–0.0006). The vertical C/D ratio was correlated positively with the IOP (ρ = 0.483, *P*<0.0001) and negatively with the CRAE, cpRNFLT, and MD (ρ = -0.745–-0.479, *P*<0.0001–0.0001). The number of medications was correlated positively with the IOP, vertical C/D ratio, ACF (ρ = 0.370–0.411, *P* = 0.0011–0.0036) and negatively with the MD, cpRNFLT, and CRAE (ρ = -0.523–-0.390, *P*<0.0001–0.0021).

**Table 2:** Correlation between various parameters.

ρ* p*	log MAR VA	Axial length	IOP	MD	v C/D ratio	cpRNFLT	mIRT	CRAE	CRVE	ACF	CECD	Medications Used
log MAR VA		0.0258	0.0494	-0.3514	0.2248	-0.1332	0.0771	-0.3275	-0.3377	0.4793	0.0396	0.068
Axial length	0.8446		0.1922	-0.1287	0.0708	-0.2596	-0.0986	-0.0700	-0.0358	0.0607	-0.0401	0.1769
IOP	0.7079	0.1412		-0.4737	0.4829	-0.5617	-0.2906	-0.4314	-0.405	0.2407	-0.2441	0.3903
MD	0.0059	0.327	0.0001		-0.6585	0.7449	-0.1722	0.4990	0.1928	-0.4711	.3165	-0.5299
v C/D ratio	0.0842	0.591	< .0001	< .0001		-0.7543	0.2192	-0.4792	-0.1929	0.3038	-0.2624	0.3703
cpRNFLT	0.3102	0.0452	< .0001	< .0001	< .0001		0.0275	0.4564	0.3684	-0.32	0.2807	-0.4918
mIRT	0.558	0.4533	0.0243	0.1883	0.0924	0.8349		0.3826	0.2358	0.1872	-0.2045	0.1572
CRAE	0.0106	0.5953	0.0006	< .0001	0.0001	0.0002	0.0026		0.4469	-0.3961	0.1378	-0.3898
CRVE	0.0083	0.7857	0.7584	0.1399	0.1397	0.0038	0.0697	0.0003		-0.5175	-0.1440	-0.2315
ACF	0.0001	0.6451	0.0639	0.0001	0.0183	0.0127	0.152	0.0017	< .0001		-0.0658	0.4106
CECD	0.7636	0.7611	0.0601	0.0137	0.0428	0.0298	0.1171	0.2937	0.2722	0.6174		-0.0844
Medications Used	0.6058	0.1764	0.0021	< .0001	0.0036	< .0001	0.2302	0.0021	0.0751	0.0011	0.5215	

PE, pseudoexfoliation material; log MAR VA, logarithm of the minimum angle of resolution visual acuity; IOP, intraocular pressure; MD, visual field mean deviation values; vC/D ratio, vertical cup-to-disc ratio; cpRNFL, circumpapillary retinal nerve fiber layer; mIR, macular inner retinal; CRAE, central retinal arteriolar equivalent; CRVE, central retinal venular equivalent; ACF, anterior chamber flare; CECD, corneal endothelial cell density; Medications, number of antiglaucoma medications use.

*Correlation coefficient (ρ) and P values for each pair of parameters are calculated using Spearman’s correlation test.

To assess possible associations between the glaucoma severity and retinal vessel narrowing, we compared the CRAE between the PE+ and PE- eyes in two subgroups based on the difference in the magnitude between both eyes, i.e., less than 5 decibels (dB) and 5 or more dB of visual field damage (Table 3). As a result, the CRAE was significantly (*P*<0.01 for both comparisons) smaller in the PE+ eyes than in the PE- eye in both subgroups.

**Table 3:** Comparison of CRAE between PE (+) and PE (-) eyes in visual field damage in two subgroups based on the magnitude of the difference between both eyes in visual field damage.

	PE (+)	PE (-)	P value*
Subjects with smaller difference between both eyes in visual field damage (MD < 5 dB) (μm)	116.9 ± 11.2	122.0 ± 9.6	0.0098
(n = 9)			
Subjects with lager difference between both eyes in visual field damage (MD ≥ 5 dB) (μm)	115.4 ± 11.4	126.8 ± 10.7	< .0001
(n = 21)			

PE, pseudoexfoliation material; MD, visual field mean deviation values.

*By the Wilcoxon signed-rank test.

Data are expressed as mean±standard deviation.

## Discussion

Based on the comparison of various ocular parameters between the PE+ and PE- eyes of patients with unilateral PEXin the current study, we found that both the central retinal arterioles and venules were significantly smaller in PE+ eyes than in fellow PE- eyes. In PE+ eyes, we also confirmed a significantly higher IOP and lower MD values suggested the presence of severer glaucoma [22], poorer logMAR VA suggested progressed cataract [23], higher ACF suggested blood-aqueous barrier breakdown[24, 25] and lower CECD suggested keratopathy [26] in PE+ eyes as reported previously.

Previous studies have reported reduced blood flow in the optic disc and ocular artery in PEX[10, 27]; however, few studies have focused on the retinal vessel diameter. Thus, the interocular comparison of the retinal vessel diameters in PE+ and PE- eyes in the same patients with clinically unilateral PEX is unique in the literature. Previously, the RNFL thickness has been reported to be significantly thinner in PEX than in a normal an age-matched control group, although no significant difference in the retinal vessel diameter was found between the two groups[28]. The vessel diameters are affected by systemic factors including various systemic diseases and medications. Thus, these systemic factors might bias a comparison of retinal vessel diameters between individuals[29]. Accordingly, cancellation of such interindividual biases might explain the current finding of vessel narrowing in PE+ eyes.

In healthy individuals, the retinal vessel diameter was equivalent between both eyes (correlation coefficients, 0.77 for retinal arterioles and 0.70 for retinal venules)[30]. In unilateral PEX eyes, reduced blood flow in the optic nerve head and peripapillary retina was observed independent of glaucoma[31]. Other investigators have reported an association between glaucoma and retinal arteriolar narrowing[32]. Accordingly, the smaller retinal vessel diameters seen in PE+ eyes in the current study can be explained by two mechanisms, i.e., glaucoma and PE deposition. It has been hypothesized that retinal arteriolar narrowing in glaucomatous eye might reflect a secondary phenomenon via autoregulatory mechanisms caused by loss of ganglion cells and resultant reduced retinal demand for oxygen[33–35]. In the current study, 27 (90%) patients had glaucoma; the CRAE, cpRNFL, and MD were correlated positively with each other (ρ = 0.469–0.745, *P*<0.0001–0.0001). Thus, glaucoma progression might directly affect the retinal arteriolar narrowing. Yuksel and colleagues have found a significantly thinner RNFL in the PE+ eye than PE- eye in a unilateral PEX without glaucoma, and they suggested an ocular blood flow disturbances by PE deposition might contribute to the development of inner retinal atrophy [36]. Besides, subgroup analysis showed an intereye difference in the CRAE irrespective of the intereye difference in magnitude in the visual field defects (Table 3), suggesting a complex association between glaucoma severity and vessel narrowing. Therefore, glaucoma was not likely the sole factor responsible for vessel narrowing in PE+ eyes.

We observed smaller vessel diameters in the arterioles and venules in PE+ eyes. In glaucomatous eyes, retinal arteriolar narrowing was not accompanied by venular narrowing[15, 37] and the RNFL thickness was correlated significantly with the CRAE but not with the CRVE[35]. A smaller retinal arteriolar diameter was associated with the incidence of OAG after adjusting for glaucoma risk factors, whereas a smaller venular diameter was not[38]. The discrepancy between the changes in the retinal arteriolar and venular diameters in patients with glaucoma has been hypothesized to result from clinically asymptomatic engorgement of the retinal veins resulting from the glaucomatous modification of the lamina cribrosa and a different regulatory mechanism[39]. Collectively, previous reports have suggested that glaucoma itself is associated more closely with the retinal arteriolar narrowing than the retinal venular narrowing[15, 37, 38]. Histologic studies have reported deposition of PE on both walls of the central retinal arteries and veins[11]. By the light and electron microscopic observations, Shimizu et al. have reported the thinning and degeneration of endothelium in the region of iris stromal vessel wall where PE deposited [12]. Single nucleotide polymorphisms of the *lysyl oxidase like1* (*LOXL1*) gene, the transcripts responsible for cross-linking of elastin, were associated highly with PEX[40, 41]. *LOXL1* expresses various ocular tissues including the endothelial cells of the conjunctiva, and the intra- and episcleral, iridal, ciliary, choroidal, retinal, and optic nerve blood arteries and veins[42]. Using the ultrasound wall-tracking system, lower distensibility and higher rigidity of the common carotid artery were detected in subjects with PEX/PEX glaucoma than in controls[43]. The significantly higher ACF in the current PE+ eyes suggested an association between PE and blood-aqueous barrier breakdown[24, 25]. In addition, negative correlation between ACF and CRAE (ρ = -0.396, *p* = 0.0017), CRVE (ρ = -0.517, *P*<0.0001) suggested that ACF might reflect the endothelial disturbance of ocular vessels. Furthermore, positive correlation between CRAE and CRVE (ρ = -0.4469, *p* = 0.0003) suggested that PE deposition likely is associated with vascular dysregulation in both the arteries and veins due to increase in retinal vessel stiffness and weaken or failed autoregulation which is the ability of an organ to maintain a constant local blood flow despite fluctuations in blood pressures or IOP. Thus negative correlation between CRAE and IOP (ρ = -0.431, *p* = 0.0006) might reflect the disturbed autoregulation against IOP elevation.

In the current study, medication was negatively correlated with CRAE (ρ = -0.390, *p* = 0.0021), thus antiglaucoma eye drops might affect the retinal vessel diameters. However, previous report did not detect any change of the retinal arterial diameter after the instillation of beta-blockers or latanoprost [44], conversely, these antiglaucoma eye drops were reported to increase ocular blood flow by IOP reduction[45, 46]. Since the MD values were negatively correlated with medications (ρ = -0.530, *p*<0.0001) and positively correlated with CRAE, narrower CRAE with greater number of medications seems to be explained by the function of glaucoma severity (i.e. eyes with higher IOP required more medications) rather than the direct effect of medications on retinal artery diameter.

The retrospective nature of the data acquisition and small sample size might be associated with a selection bias. Because of the retrospective study design, we could not consider the blood pressure, detailed systemic diseases, and systemic medications, which might have affected the retinal vessel diameter. However, the effects of these factors should have been canceled by the comparison between eyes of a subject in this study. In the current study, various diagnostic glaucoma parameters were correlated significantly with the CRAE and CRVE. Among them, negative correlations between the CRAE and cpRNFL and between the CRAE/CRVE and ACF were unique findings.

## Conclusions

Deposition of PE can cause retinal vessel narrowing in arterioles and venules. The roles and mechanisms of retinal vessel narrowing in glaucoma pathogenesisneeds clarification.

## Supporting information

S1 File Medical dataset of patients. (XLSX)
